# Cell Proliferation in Neuroblastoma

**DOI:** 10.3390/cancers8010013

**Published:** 2016-01-12

**Authors:** Laura L. Stafman, Elizabeth A. Beierle

**Affiliations:** Department of Surgery, Division of Pediatric Surgery, University of Alabama, Birmingham, AL 35233, USA; lstafman@uabmc.edu

**Keywords:** neuroblastoma, *MYCN*, kinases, cell cycle check point inhibitors

## Abstract

Neuroblastoma, the most common extracranial solid tumor of childhood, continues to carry a dismal prognosis for children diagnosed with advanced stage or relapsed disease. This review focuses upon factors responsible for cell proliferation in neuroblastoma including transcription factors, kinases, and regulators of the cell cycle. Novel therapeutic strategies directed toward these targets in neuroblastoma are discussed.

## 1. Neuroblastoma

Neuroblastoma, a tumor of neural crest origin, is the most common extracranial solid tumor of childhood, accounting for 7% of childhood malignancies and 15% of childhood cancer mortality [1,2]. More than 50% of these tumors occur in children less than 2 years of age. The incidence of neuroblastoma has increased in recent years and it continues to carry a poor prognosis in children over two years of age with a survival of only 38% [3,4]. Overall 5-year survival was 74% from 1999–2005 [5]. Neuroblastoma exhibits a wide array of biological characteristics and behaviors, which are important in predicting outcomes.

## 2. Cell Proliferation

Cell proliferation refers to an increase in the number of cells due to cell growth and division and is necessary not only for growth and normal tissue function but also tumorigenesis. From embryological development through senescence, cell proliferation is maintained by a strict coordination of cellular signals and deregulation of cell proliferation is the defining feature of all tumors. The mechanisms of deregulation vary but are inevitably via perturbations in signal transduction within the cell. Many of these perturbations are in signaling pathways important for proliferation—those regulating cellular growth, differentiation, and developmental signals—and are the result of mutations, amplifications, gene overexpression, or chromosomal deletions [6]. Therefore, manipulation of cell proliferation pathways may decrease the malignant potential of tumors, including neuroblastoma, making these pathways an attractive target for novel therapeutics. Manipulation of the pathways involved in cell differentiation can also decrease the malignant potential of neuroblastoma. Differentiation and proliferation are separate but concurrent processes that are regulated independently by signaling pathways, although some of the pathways overlap and crosstalk [7]. This review will discuss some of the common cell proliferation proteins and pathways involved in neuroblastoma, including transcription factors, kinases, and regulators of the cell cycle.

## 3. Neuroblastoma and Transcription Factors

Multiple transcription factors, which initiate and regulate gene transcription, have been implicated in neuroblastoma pathogenesis via an increase in cell proliferation. These include N-MYC, nuclear factor kappa-light-chain-enhancer of activated B cells (NF-κB), paired-like homeobox 2b (PHOX2B), and P53.

*MYCN* oncogene encodes N-MYC, a phosphoprotein in the MYC family of transcription factors. N-MYC binds to the E-box sequences CACGTG and CATGTG predominantly in the *MYCN*-amplified state [8,9]. When bound to these promoter sequences, N-MYC acts as a classical transcription factor, increasing expression of individual genes that increase cell proliferation, and changes global chromatin structure by DNA hypermethylation, which also increases cell proliferation. N-MYC is expressed early in development up through a few weeks following birth [10]. After that, it is only found in adult B cells. *MYCN* amplification is detected in approximately 20% of neuroblastoma tumors [11,12], and amplification of greater than ten copies of *MYCN* is the strongest adverse prognostic indicator for this disease [13]. The significance of *MYCN* amplification in neuroblastomas was first noted in the 1980s [13,14]. More recently, Molenaar *et al.* found that the majority of high-risk neuroblastoma tumors had genomic amplifications of *LIN28B*, which suppresses LET-7 and resulted in increased N-MYC protein expression [15].

Using *in vitro* studies, it has been demonstrated that the level of N-MYC expression correlates with cell proliferation in neuroblastoma [16,17]. N-MYC has been shown to increase cell proliferation in neuroblastoma via multiple mechanisms. Transient receptor potential cation channels M6 and M7 (TRPM6 and TRPM7) are transcriptional targets of N-MYC. These proteins promote cellular calcium and magnesium uptake and enhance cell proliferation in neuroblastoma [18]. N-MYC upregulates high mobility group protein of the A type 1 (HMGA1), an important regulator of cell growth which also leads to increased cell proliferation [19]. E2F2 is transcriptionally activated by N-MYC and induces transcription activation and progression through the cell cycle, with the ultimate effect of increasing cell proliferation [20,21]. N-MYC also upregulates serine hydroxymethyltransferase 2 (SHMT2), which breaks down serine into glycine and a one-carbon moiety. The glycine produced is an important source of methyl groups for biosynthesis, and as such, SHMT2 leads to increased cell proliferation. Using a panel of diverse human cancer cell lines, it was demonstrated that glycine consumption correlated with rapid cell proliferation in cancer cells [22]. SHMT2 has been found to be highly expressed in aggressive *MYCN*-amplified neuroblastomas [23]. MYC also regulates transcription of PHGDH, an enzyme upstream in the SHMT2 pathway that converts 3-phosphoglycerate to serine. Six of the eleven genes in this pathway have been identified as targets of MYC [24]. The protein sulfatase 2 (SULF-2) is overexpressed in *MYCN*-amplified neuroblastoma cells but expressed at a much lower level in *MYCN*-unamplified cells [25]. Suppression of SULF-2 using small interference RNA (siRNA) decreased cell proliferation in *MYCN*-amplified neuroblastoma cells, indicating that SULF-2 is a potential drug target. A SULF-2 small molecule inhibitor, OKN-007, has been developed, but has yet to be examined in neuroblastoma.

Many researchers have targeted *MYCN* transcription in the development of anti-tumor therapies. Using antisense oligonucleotides directed against human *MYCN* in *MYCN*-amplified human neuroblastoma cells yielded approximately half as much N-MYC protein expression compared to control and *in vivo* led to decreased tumor growth in transgenic *MYCN* mice [26]. Antisense oligonucleotides are quickly degraded by nucleases, limiting their clinical potential, but more recently, morpholino antisense oligomers have been developed. These are more stable and function to knock down genes by modifying the splicing of pre-mRNA. However, to our knowledge, these have not yet been utilized to target *MYCN* in neuroblastoma. Kang *et al.* used siRNA directed against *MYCN* and found a downregulation in N-MYC protein expression in *MYCN*-amplified neuroblastoma cells but not in *MCYN* unamplified cells [27]. Additionally, proliferation in *MYCN*-amplified neuroblastoma cells was decreased with si*MYCN* treatment. The delivery method to the target cells and off-target effects in the clinical setting remain challenges for the clinical application of siRNA technology. An anti-gene peptide nucleic acid (PNA) conjugated with a nuclear localization signal peptide targeted against a sequence of *MYCN* DNA was determined to decrease N-MYC expression. Additionally, in *MYCN-*amplified and *MYCN*-unamplified/low-expressed human neuroblastoma cell lines cell proliferation decreased significantly, but in *MYCN*-unamplified/unexpressed cells there was no decrease in cell proliferation [28]. PNA therapy is promising given the strong binding between PNA and DNA, but PNAs are hydrophobic and difficult to deliver to target cells. MicroRNAs (miRNAs) occur naturally in humans and regulate expression of genes by binding to mRNAs and inhibiting protein production. Using miRNAs targeted against *MYCN* mRNA, Buechner and colleagues noted reduced endogenous N-MYC expression and significantly impaired proliferation in *MYCN*-amplified neuroblastoma cell lines [29]. In contrast to siRNAs, miRNAs’ complementarity to the target mRNA is not exact, so each miRNA may target many different mRNAs and concerns remain regarding off-target effects. Recently, Puissant showed that bromodomain inhibition suppressed *MYCN* transcription in neuroblastoma [30]. Using a novel ligand, JQ1, which competitively displaced bromodomain and extra-terminal domain (BET) bromodomains from chromatin, *MYCN* transcription was decreased *in vitro*. Additionally, in a *MYCN*-amplified neuroblastoma murine model, JQ1 treatment significantly decreased tumor volume and increased survival compared to controls. Other BET bromodomain inhibitors are in preclinical trials including I-BET762 [31]. OTX015 is a BET bromodomain inhibitor which decreased cell proliferation in *MYCN*-amplified neuroblastoma cells in preclinical trials and is currently in Phase I clinical trials for other solid tumors [32].

Other researchers have targeted the N-MYC protein. In order for N-MYC to act as a transcription factor, it must localize to the nucleus and dimerize with MAX protein. This dimer then binds to a specific E-box sequence in DNA to transcribe genes important for cell proliferation. However, when cells have higher levels of MAX, there is not enough N-MYC available to form heterodimers, so MAX homodimerizes. Instead of stimulating proliferation, MAX homodimers stimulate transcription of genes important for cell differentiation [33]. An inhibitor of N-MYC/MAX dimerization, 10058-F4, caused cell cycle arrest, apoptosis, and differentiation *in vitro*, and increased survival in *MYCN* transgenic mice [34]. MAD is another protein that forms heterodimers with MAX. However, as opposed to N-MYC, which is expressed in proliferating tissues, MAD is a protein expressed in differentiated tissue. When MAD forms a heterodimer with MAX, it binds to the same E-box sequence, but this dimer represses transcription, thereby inhibiting cell proliferation [35,36].

NF-κB is another important transcription factor that may be activated in neuroblastoma [37]. In cells other than B cells and macrophages, NF-κB is sequestered in the cytoplasm through an interaction with inhibitor of κB (IκB) proteins [38]. It is not until IκB is phosphorylated that NF-κB is free to translocate to the nucleus where it binds to promoter sequences to induce transcription [39]. When phosphorylated, IκB is ubiquitinated and then degraded by proteasomes. Bortezomib is a reversible inhibitor of the 26S proteasome [40]. By inhibiting the proteasomal degradation of a host of proteins including IκB, bortezomib decreased cell proliferation in neuroblastoma cells and decreased growth and the number of mitotic cells in murine neuroblastoma models [41,42,43]. A phase I clinical trial found that bortezomib was well-tolerated in children with recurrent or refractory solid tumors, two of whom had neuroblastoma [37,44]. In some forms of neuroblastoma, NF-κB has been shown to lead to increased migration and invasion but in others it induced apoptosis [45,46]. Curcumin suppresses NF-κB activity, and was shown to inhibit proliferation in neuroblastoma cell lines and decrease tumor growth and cell proliferation in a murine neuroblastoma model [47]. Gao *et al.* used oleanolic acid derivatives, CDDO-Im and CDDO-Me, for NF-κB inhibition and saw decreased neuroblastoma cell proliferation [48]. CDDO-Me has also been demonstrated to delay tumor growth in neuroblastoma xenografts [49].

A third transcription factor, PHOX2B, is expressed exclusively in the nervous system. Germline mutations of *PHOX2B* predispose to hereditary neuroblastoma and have also been observed in sporadic cases [50,51,52,53], but remain a rare cause of neuroblastoma [54]. Sympathetic neural cells derived from chick and mouse embryos transfected with gain-of-function PHOX2B variants seen in neuroblastoma demonstrated significantly more cell proliferation than wild-type PHOX2B, indicating that wild-type PHOX2B may act a tumor suppressor [55]. PHOX2B has not yet been targeted for development of therapeutics given the low frequency of involvement in neuroblastoma.

Mutations in the tumor suppressor, P53, are the most frequent genetic alterations found in human cancer [56]. Mutations in P53 are infrequent in neuroblastoma and are mostly limited to relapsed tumors [57,58,59,60], but wild-type P53 is often destabilized and not functional. In undifferentiated neuroblastomas, wild-type P53 was sequestered in the cytoplasm preventing its function as a transcription factor [61]. N-MYC has been found to upregulate P53 expression in neuroblastoma [62], but the protooncogene MDM2 which negatively regulates P53 is a direct transcriptional target of N-MYC, leading to increased MDM2 and subsequent decreased P53 function in *MYCN*-amplified neuroblastoma cells [63]. Nutlin-3, an MDM2 antagonist, releases P53 from negative control by MDM2 and thus stabilizes and activates P53. Using an *in vitro* model of acquired drug resistance in neuroblastoma, van Maerken treated cells with nutlin-3 and saw a significant decrease in cell proliferation in cell lines with wild-type P53 but not in those with mutant P53 [64]. They also noted increased expression of P53 and its target genes, indicating that P53 activation in neuroblastoma by nutlin-3 may yield a new therapy for drug-resistant neuroblastoma with wild-type P53. Additionally, *MYCN*-amplified neuroblastoma cells treated with MDM2 antagonists, nutlin-3 and MI-63, had decreased cell proliferation when compared to *MYCN*-unamplified cells, further illuminating the importance of the relationship between *MYCN* and P53 in neuroblastoma [65]. A summary of the drugs discussed in this section is provided in Table 1.

**Table 1:** Proteins that affect proliferation in neuroblastoma through transcription and their targeted drugs. Mechanism of action and stage of development in neuroblastoma is listed along with references.

Target Protein	Drug	Mechanism of Action	Stage of Development	References
SULF-2	si*SULF-2*	Double-stranded RNA that cleaves *SULF-2* mRNA	*In vitro*	[25]
N-MYC	si*MYCN*	Double-stranded RNA that cleaves *MYCN* mRNA	*In vitro*	[27]
	PNA	Synthetic polymer that binds *MYCN* DNA to inhibit N-MYC expression	*In vitro*	[28]
	miRNAs	Single-stranded RNA that inhibits translation of *MYCN* mRNA	*In vitro*	[29]
	JQ1	Inhibits BET bromodomains	*In vivo*	[30]
	I-BET762	Inhibits BET bromodomains	*In vivo*	[31]
	OTX015	Inhibits BET bromodomains	Clinical trial	[32]
	10058-F4	Inhibits N-MYC/MAX dimerization	*In vivo*	[34]
NFκB	Bortezomib	Inhibits proteasomal degradation of IκB, thus deactivating NFκB	Clinical trial	[40,41,42,43,44]
	Curcumin	Inhibits activation of NFκB	*In vivo*	[47]
	Oleanolic acid derivatives (CDDO-Im and CDDO-Me)	Inhibit TNFα-induced targeting of NFκB to the nucleus	*In vivo*	[48,49]
MDM2	Nutlin-3	Releases P53 from negative control by MDM2	*In vivo*	[64]
	MI-63	Releases P53 from negative control by MDM2	*In vitro*	[65]

## 4. Neuroblastoma and Kinases

Kinases are proteins defined by their ability to phosphorylate other proteins in cell signaling pathways. Phosphorylation either activates or inactivates the downstream protein, which in turns affects the signaling cascade. In neuroblastoma, many kinases have been found to play a role in cell proliferation.

One of the well-described signaling pathways is the RAS/RAF/MEK/ERK cascade. RAS is a small GTPase that is activated when a mitogen binds to a membrane receptor tyrosine kinase. A serine/threonine kinase, RAF, is then targeted to the cell membrane, interacts with RAS, dimerizes, and undergoes phosphorylation for activation. RAF phosphorylates MEK and MEK in turn phosphorylates ERK. Once activated by phosphorylation, ERK phosphorylates a number of proteins involved in proliferation. Abnormal activation of this pathway has been seen in neuroblastoma. A recent study found that the majority (78%) of relapsed neuroblastomas had mutations predicted to activate the RAS/RAF/MEK/ERK pathway, but very few primary tumors had these mutations [66]. In preclinical studies, treatment with the MEK inhibitor U0126 decreased proliferation in neuroblastoma cell lines with high constitutive total ERK, but not in those with less total ERK, indicated that this pathway plays a role in cell proliferation in some forms of neuroblastoma [67]. Treatment of RAS/RAF-mutated neuroblastoma cell lines with the MEK inhibitors trametinib, cobimetinib, and binimetinib also decreased cell growth [66]. MEK inhibitors warrant further study as targeted therapies for relapsed neuroblastoma.

The phosphoinositide 3-kinase (PI3K)/Protein kinase B (AKT) cascade has been implicated in neuroblastoma [68,69,70]. When activated, PI3K phosphorylates phosphatidylinositol to produce a secondary messenger that binds downstream targets, including AKT, recruiting them to the cell membrane. The serine/threonine kinase, AKT, then acts on downstream targets to promote cell survival and proliferation. Via a series of intermediaries, the serine/threonine kinase, mechanistic target of rapamycin (mTOR), is activated [71]. Neuroblastoma tissue was found to have significant levels of activated AKT and mTOR compared to the normal adrenal medulla [68,72]. Multiple human neuroblastoma cell lines including SK-N-SH, SK-N-BE, and IMR-32 have been found to have activated AKT. Activated AKT correlated with advanced stage, unfavorable histology, poor outcome, and *MYCN* amplification in human tumor specimens [70]. Inhibition of mTOR with rapamycin and an analog, CCI-779, decreased neuroblastoma cell proliferation, indicating a role for the PI3K/AKT/mTOR pathway in tumorigenesis [68]. There are conflicting data regarding the relationship between *MYCN* amplification and the PI3K/AKT/mTOR pathway. Johnsen *et al.* found that the decrease in cell proliferation in *MYCN*-amplified tumors was greater than that observed in *MYCN* non-amplified tumors [68], but Misawa *et al.* found that maintenance of cell proliferation in neuroblastoma required mTOR function, but it appeared to be independent of *MYCN* induction [69].

There are a number of drugs targeting the PI3K/AKT/mTOR cascade currently in development. mTOR inhibitors rapamycin (also termed sirolimus) and its analogues, including temsirolimus, everolimus, and ridaforolimus function by binding to the cytosolic protein FK-binding protein 12 (FKBP12). Once bound to FKBP12, the complex binds directly to mTOR, inhibiting its action [73]. Rapamycin is currently in clinical trials and has been demonstrated to decrease cell proliferation in neuroblastoma [68,69,74]. However, in neuroblastoma cells, rapamycin introduced potential for resistance by way of inducing expression of survivin, a protein that protects cells from apoptosis [74]. A phase II study of temsirolimus in children with relapsed or refractory high-grade neuroblastoma was halted as there were no objective responses. In that study it was noted that 32% of neuroblastoma subjects did experience disease stabilization, so temsirolimus may be used in the future in combination with other therapies [75]. A phase I study of everolimus in neuroblastoma has been completed and the drug is well-tolerated in children, but no objective responses were reported [76]. Similar findings were observed in a phase I study of ridaforolimus in children with refractory solid tumors [77]. It is clear that rapamycin and its analogues have only modest activity against neuroblastoma, but their effectiveness as combination therapies remains to be elucidated.

mTOR inhibitors that act as ATP-competitive inhibitors are also being evaluated. These include INK128/MLN0128, AZD2014, and OSI027. In both *MYCN*-amplified and non-amplified neuroblastoma cell lines, treatment with INK128/MLN0128 suppressed cell proliferation. Using xenograft models of neuroblastoma, INK128/MLN0128 led to significant tumor growth inhibition in one study, but failed to prevent progressive disease in another, although some tumors did exhibit significantly less growth [78,79]. AZD2014 has shown promise in breast cancer [80] and OSI027 had anti-proliferative activity in several human cancer cell lines, but they have not yet been examined in neuroblastoma [81]. Similar to rapamycin, the ATP-competitive mTOR inhibitors appear to have limited activity against neuroblastoma cell proliferation.

There is a feedback loop that causes reactivation of PI3K and the RAS/RAF/MEK/ERK cascade when mTOR activity is decreased [82]. Given the modest activity of both groups of mTOR inhibitors against neuroblastoma and this feedback loop, researchers have turned to dual inhibitors targeting both PI3K and mTOR. NVP-BEZ235 is one such compound that has been used *in vitro* and found to have an anti-proliferative effect in neuroblastoma cell lines [83]. In an *in vivo* neuroblastoma model with TH-*MYCN* mice, NVP-BEZ235 suppressed proliferation of tumor cells and angiogenesis [84]. SF1126, another dual PI3K/mTOR inhibitor, has been demonstrated to inhibit neuroblastoma cell proliferation [85]. NVP-BKM120, developed with a different profile of targets within the PI3K/mTOR family, also inhibited cell proliferation in a variety of tumor cells, but preferentially inhibited tumor cells with *PIK3CA* oncogenic mutations [86]. This mutation is rare in neuroblastoma; Dam *et al.* found *PIK3CA* mutations in only 2.9% of 69 human neuroblastoma samples [87], limiting its utility for neuroblastoma. Finally, GDC-0980, has been shown to decrease cell proliferation in a variety of adult cancer cell lines, but has not been studied in neuroblastoma [88]. All of these dual PI3K/mTOR inhibitors are in various stages of clinical trials.

Another mechanism to circumvent the feedback loops that occur with mTOR inhibition is targeting the AKT protein upstream from mTOR. MK-2206 is an AKT inhibitor that has been shown to inhibit cell proliferation in neuroblastoma cells and inhibit tumor growth and increase survival in mice bearing xenograft neuroblastoma tumors [89]. A phase I trial of MK-2206 that included 3 children with neuroblastoma found that the drug was well-tolerated, but there were no objective responses or prolonged stabilization of disease [90]. Perifosine, an AKT inhibitor that also inhibits the ERK and JNK pathways [91,92], decreased neuroblastoma cell proliferation, decreased tumor growth, and increased survival in mice bearing neuroblastoma xenografts [93]. Perifosine is currently in phase I trials in pediatric solid tumors.

RET is a receptor tyrosine kinase required for normal development of the nervous system that has been found to be expressed in most neuroblastomas and overexpressed in some [94]. Upon addition of glial cell line-derived neurotrophic factor (GDNF), a growth factor and ligand for RET that neuroblastoma cells secrete, cell proliferation was stimulated in non-adherent neuroblastoma cells but not in adherent cells. These findings indicated that RET/GDNF played a role in cell proliferation in neuroblastoma [95]. Additionally, a RET-overexpressing transgenic mouse spontaneously developed neuroblastoma [96]. Vandetanib/ZD6474 inhibits activation of RET and was found to decrease neuroblastoma cell viability and proliferation and inhibit growth in neuroblastoma xenografts [97,98].

Another receptor tyrosine kinase implicated in neuroblastoma is CD117, also known as C-KIT and mast/stem cell growth factor receptor. CD117 has been correlated with both favorable and unfavorable tumors [99,100]. Vitali *et al.* found that 13% of neuroblastomas expressed CD117 and 23% expressed its ligand, stem cell factor (SCF), and *MYCN*-amplified tumors were more likely to express CD117 and SCF than non-amplified tumors. CD117 stimulation actively promoted cell proliferation in neuroblastoma [100]. Another group showed that 45% of human tumor samples simultaneously expressed both CD117 and SCF [101]. Imatinib, a selective inhibitor of tyrosine kinases including CD117, has been demonstrated to inhibit proliferation in neuroblastoma cells [100]. Additionally, in a murine xenograft model, animals treated with imatinib developed smaller tumors than controls [102]. The concentration of imatinib required to achieve growth inhibition in neuroblastoma was higher than that required to inhibit CD117 activation indicating that there was an additional mechanism of action. In neuroblastoma cells, imatinib was also found to inhibit C-ABL, a tyrosine kinase implicated in multiple cancers due to its role in cell differentiation, proliferation, and adhesion. Imantinib-induced C-ABL inhibition yielded a dose-dependent decrease in neuroblastoma cell proliferation [103]. A phase II clinical trial studying imatinib in refractory and relapsing metastatic neuroblastoma found that it was well-tolerated and effective with 21% showing a complete response and 8% a partial response [104].

Neuroblastoma cells have been shown to have significantly higher activity of the kinase C-SRC than normal human fibroblasts or glioblastoma cells [105]. Expression of C-SRC is correlated with a favorable prognosis and is inversely correlated with *MYCN* amplification [106]. Investigators have targeted C-SRC in an attempt to treat neuroblastoma. The C-SRC inhibitor PP2 and the dual C-SRC/ABL inhibitor dasatinib decreased cell proliferation in neuroblastoma cells [107,108]. As selective C-SRC inhibitors continue to be developed, their anti-proliferative activity improves, making them a potential treatment for neuroblastoma [109].

Focal adhesion kinase (FAK) is a non-receptor tyrosine kinase that is regulated by N-MYC and is more frequently expressed in aggressive forms of neuroblastoma [110]. When phosphorylated, FAK binds to SRC and activates downstream signaling pathways that control cell proliferation, viability, motility, and survival [111,112,113,114]. Inhibition of FAK has been achieved using a variety of methods. By using adenoviral gene transduction of the carboxyl-terminal domain of FAK (AdFAK-CD), FAK phosphorylation was decreased [115] and AdFAK-CD decreased neuroblastoma cell proliferation, with a larger proportional decrease in proliferation of *MYCN*-amplified cells than *MCYN* non-amplified cells [116]. siRNA has also been used to decrease expression of FAK. In neuroblastoma cells, treatment with siFAK led to decreased cell survival, with a larger effect in *MYCN*-amplified cell lines [117]. Small molecule inhibitors of FAK include NVP-TAE226, 1,2,4,5-benzenetetramine tetrahydrochloride (Y15), and PF-04554878. Treatment of neuroblastoma cells with NVP-TAE226 resulted in a decrease in cell viability [118]. Y15 has been demonstrated to decrease viability in neuroblastoma cells and to inhibit neuroblastoma tumor growth in mouse xenograft models [119]. A phase I clinical trial studying the small molecular FAK inhibitor, PF-04554878, in adults with advanced solid tumors has been completed and others are currently in progress. Another approach to inhibiting FAK is by interrupting its interaction with vascular endothelial growth factor receptor 3 (VEGFR-3) using a small molecular inhibitor, chloropyramine hydrochloride (C4). Treatment of neuroblastoma cells with C4 decreased cell viability and treatment of mouse xenografts with C4 decreased neuroblastoma tumor growth [120].

Aurora kinase A is a serine/threonine kinase that has been associated with poor prognosis in neuroblastoma [121]. Aurora kinase A stabilizes N-MYC, thereby increasing neuroblastoma cell proliferation [122,123]. Additionally, Aurora kinase A activity increased VEGF secretion and angiogenesis. Inhibition of Aurora A kinase with the small molecule alisertib/MLN8237 decreased cell proliferation and inhibited anchorage-independent growth in neuroblastoma cells [124]. A structurally similar inhibitor, MLN8054, was shown to inhibit proliferation in neuroblastoma cells and both MLN8054 and alisertib/MLN8237 led to complete response rates of about 50% in a transgenic TH-*MYCN* mouse model of neuroblastoma [123]. In a phase I/II clinical trial for MLN8237 in children with refractory/recurrent solid tumors 2 of 11 children with neuroblastoma attained prolonged stable disease [125]. Aurora kinase A inhibitors offer a promising new direction for treatment of neuroblastoma, particularly in *MYCN*-amplified neuroblastomas given the relationship between Aurora kinase A and N-MYC.

Aurora kinase B is a related serine/threonine kinase that plays a role in the attachment of the mitotic spindle to the centromere during mitosis. Patients with high Aurora kinase B expression have a worse prognosis than those with low/normal expression [126]. Aurora kinase B does not stabilize N-MYC and does not affect N-MYC levels [123]. Conversely, it is transcriptionally regulated by N-MYC. Barasertib is a specific Aurora kinase B inhibitor which has been shown to decrease cell growth in neuroblastoma cell lines which are *MYCN*-amplified and have wild-type P53 [126]. However, *MYCN-*unamplified and mutant P53 cell lines were less sensitive to the drug. Using the IMR5 neuroblastoma cell line, which is *MYCN*-amplified and has wild-type P53, barasertib induced cell cycle arrest at G_2_/M. In a xenograft model with the same cell line, barasertib decreased tumor growth. Barasertib has not yet been examined in pediatric clinical trials. CCT137690, an inhibitor of both Aurora kinase A and B, inhibited cell proliferation in *MYCN*-amplified neuroblastoma cells and tumor growth in *MYCN*-driven transgenic tumors *in vivo* [127].

Anaplastic lymphoma kinase (ALK) is a receptor tyrosine kinase expressed in the developing nervous system [128]. Oncogenic mutations in the *ALK* gene play a role in neuroblastoma pathogenesis and are highly correlated with *MYCN* amplification [129]. Mutant ALK in neuroblastoma has increased kinase activity compared to wild-type ALK. Using RNA interference (RNAi) to knock down *ALK* in neuroblastoma cells harboring mutant *ALK* resulted in decreased cell proliferation. Crizotinib/PF-02341066 is a small molecule inhibitor targeting ALK and other receptor tyrosine kinases, MET and ROS1, that decreased proliferation in neuroblastoma cell lines with abnormal ALK, particularly those with the R1275Q mutation [130]. In mice with neuroblastoma xenografts containing R1275Q-ALK, crizotinib caused tumor regression within three weeks and complete regression over the fourth week. Crizotinib is FDA-approved for treatment of non-small cell lung cancer. It has also been examined in a phase I trial in pediatric patients with refractory solid tumors including 34 patients with neuroblastoma. Crizotinib was well-tolerated with two of the 34 patients with neuroblastoma experiencing complete response and eight experiencing stable disease [131]. Multiple clinical trials are underway examining crizotinib in neuroblastoma.

Sorafenib is a multi-kinase inhibitor targeting VEGFR, PDGFR, CD117, RAF, and RET [132]. It is currently approved for treatment of renal cell carcinoma, hepatocellular carcinoma, and thyroid cancer in adults. Sorafenib has been studied in neuroblastoma and was shown to decrease cell proliferation both *in vivo* and *in vitro* with cell cycle arrest at the G_1_ checkpoint [133]. In the Pediatric Preclinical Test Program *in vitro* and *in vivo* panels, which included 4 neuroblastoma cell lines and 5 neuroblastoma xenografts, sorafenib was shown to decrease growth in the majority of neuroblastoma samples [134]. A phase I pediatric study revealed similar toxicities to those seen in adults, but no further clinical studies have been completed [135]. A summary of targets and drugs discussed in this section is available in Table 2.

**Table 2:** Kinases affecting proliferation in neuroblastoma and their targeted drugs. Mechanism of action for each drug and stage of development in neuroblastoma is listed along with references.

Target Protein	Drug	Mechanism of Action	Stage of Development	References
MEK	U0126	Binds/inhibits MEK	*In vitro*	[67]
	Trametinib	Binds/inhibits MEK	*In vitro*	[66]
	Cobimetinib	Binds/inhibits MEK	*In vitro*	[66]
	Binimetinib	Binds/inhibits MEK	*In vitro*	[66]
mTOR	Rapamycin	Binds FKBP12 inhibits mTOR	Clinical trial	[68,69,73,74]
	CCI-779/Temsirolimus	Binds FKBP12 inhibits mTOR	Clinical trial	[68,73,75]
	Everolimus	Binds FKBP12 inhibits mTOR	Clinical trial	[73,76]
	Ridaforolimus	Binds FKBP12 inhibits mTOR	Clinical trial	[73,77]
	INK128/MLN0128	ATP-competitive mTOR inhibitor	*In vivo*	[78,79]
PI3K/mTOR	NVP-BEZ235	Inhibits PI3K and mTOR	*In vivo*	[83,84]
	SF1126	Reversibly inhibits PI3K and mTOR	Clinical trial	[85,136]
	NVP-BKM120	Inhibits PI3K and mTOR in ATP-competitive manner	*In vitro*	[86]
AKT	MK-2206	Inhibits AKT via allosteric binding	Clinical trial	[89,90]
	Perifosine	Binds AKT, inhibiting translocation to the plasma membrane	Clinical trial	[93,137]
RET	Vandetanib/ZD6474	Inhibits activation of RET	*In vivo*	[97,98]
CD117/ABL/PDGFR	Imatinib	Binds active site and inhibits kinases	Clinical trial	[100,102,103,104]
C-SRC	PP2	Binds and acts as a mixed competitive inhibitor for C-SRC	*In vitro*	[107]
C-SRC/ABL	Dasatinib	Binds adenine pocket inhibits C-SRC and ABL	Clinical trial	[108,138]
FAK	AdFAK-CD	Adenoviral gene transduction of the carboxyl-terminal domain of FAK	*In vitro*	[115,116]
	si*FAK*	Double-stranded RNA cleaves *FAK* mRNA	*In vitro*	[117]
	NVP-TAE226	Inhibits phosphorylation of FAK	*In vivo*	[118,139]
	Y15	Inhibits phosphorylation of FAK	*In vivo*	[119]
	Chloropyramine hydrochloride/C4	Inhibits interaction between FAK and VEGFR-3	*In vivo*	[120]
Aurora kinase A	Alisertib/MLN8237	Binds/inhibits Aurora kinase A	Clinical trial	[123,125]
	MLN8054	Binds/inhibits Aurora kinase A	*In vivo*	[123]
Aurora kinase B	Barasertib	Binds ATP pocket/inhibits Aurora kinase B	*In vivo*	[126]
Aurora kinase A/B	CCT137690	Binds/inhibits Aurora kinase A and B	*In vivo*	[127]
ALK	si*ALK*	Double-stranded RNA cleaves *ALK* mRNA	*In vitro*	[129]
ALK/MET/ROS1	Crizotinib/PF-02341066	Binds/inhibits ALK, MET, and ROS1	Clinical trial	[130,131]
VEGFR/PDGFR/CD117/RAF/RET	Sorafenib	Binds/inhibits VEGFR, PDGFR, CD117, RAF, and RET	Clinical trial	[132,133,134,135]

## 5. Neuroblastoma and Cell Cycle Checkpoints

In normal cells, the cell cycle is highly regulated at checkpoints by multiple protein families—cyclins, cyclin dependent kinases (CDKs), CDK inhibitors (CKIs), and tumor suppressors including P53 and RB [140]. These proteins function as accelerators or brakes to advance the cell cycle to the next phase or halt the cycle at the checkpoint.

P53 regulates the G_1_/S and G_2_/M checkpoints in the cell cycle. It has been demonstrated that tumor cells lacking normal P53 function continue through the G_1_/S checkpoint. P53 is a transcription factor for WAF1/CIP1/P21, a CKI [141]. By upregulating P21 expression, CDKs are inhibited, leading to arrest of the cell cycle and therefore a decrease in cell proliferation. In some neuroblastoma cells treated with nutlin-3, a P53 activator, induction of P21 and arrest of the cell cycle arrest at the G_1_/S checkpoint was observed [65], but this effect was cell line dependent.

Another tumor suppressor, RB, has been demonstrated to play a role in neuroblastoma. It acts as a regulator of the G_1_/S checkpoint, restricting cell cycle progression and thus cell proliferation [142]. Unphosphorylated RB remains bound to the transcription factor E2F. RB phosphorylation leads to the release of E2F, which activates a multitude of proto-oncogenes and DNA polymerase to start the replication process [143,144]. In a study examining the bone marrow of 4 children with neuroblastoma, only one child had a mutation in the RB protein and that child was the only one with bone marrow infiltration who died, correlating with advanced disease [145]. RB has been indirectly targeted in neuroblastoma cells using difluoromethylornithine (DFMO). DFMO decreased RB phosphorylation in *MYCN*-amplified neuroblastoma cells and significantly decreased cell growth, likely mediated by the protein P27 [146]. To our knowledge, no drugs have been developed specifically targeting RB in neuroblastoma.

RB phosphorylation is regulated by CDK4 and CDK6. CDK4/6 mRNA expression in human neuroblastoma tissue was determined to be high compared to normal tissue [147]. CDK4 expression correlated with a poor prognosis and CDK6 expression correlated with undifferentiated histology, also correlating with poor prognosis. The specific CDK4/6 inhibitor, ribociclib/LEE011, reduced proliferation in *MYCN*-amplified neuroblastoma *in vitro* and decreased growth of murine neuroblastoma xenografts [148]. A phase I clinical trial examining LEE011 in children with malignant rhabdoid tumors and neuroblastoma is underway. Palbociclib/PD-0332991 is a CKI targeting CDK4/6 that is FDA-approved for breast cancer and has been demonstrated to effectively decrease phosphorylation of RB, causing a potent anti-proliferative effect in a variety of adult tumor cells [149]. Palbociclib has recently been found to reduce the growth of neuroblastoma cells *in vitro*, but has not yet been studied in neuroblastoma *in vivo* or in clinical trials [150].

While DFMO indirectly targets RB, it directly inhibits ornithine decarboxylase (ODC), the rate-limiting enzyme in polyamine biosynthesis [146,151,152]. Polyamines are critical for cell survival; ODC along with an increase in polyamines has been seen in a variety of cancers [153,154]. A decrease in polyamines, such as that caused by DFMO, initiates cell cycle arrest or apoptosis whereas an increase in polyamines allows the cell to continue through the cell cycle [146]. The gene expressing ODC, *ODC1*, is activated by N-MYC and is upregulated in *MYCN*-amplified human neuroblastomas [155,156]. Even some tumors without *MYCN* amplification had elevated ODC mRNA levels. High ODC expression, regardless of *MYCN* status, correlates with poor survival [156,157]. The use of DFMO in *MYCN*-amplified neuroblastoma cells has been demonstrated to deplete polyamines, impair proliferation, and induce G_1_ cell cycle arrest without apoptosis [146,158]. The cell cycle arrest was associated with an increase in the P27^Kip1^ cyclin-dependent kinase inhibitor. Treatment of neuroblastoma-prone genetically engineered TH-*MYCN* mice with DFMO suppressed neuroblastoma development [158]. A phase I trial of DFMO in children with relapsed/refractory neuroblastoma found that it was safe and tolerated well [159]. A certain subset of patients—those with a particular single nucleotide polymorphism in *ODC1* termed the minor T-allele at rs2302616—had a better response to therapy, indicating that DFMO may be useful as a targeted therapy.

Aside from CDK4 and 6, other CDKs have been examined as potential targets for neuroblastoma treatment given their importance in cell proliferation through regulation of the cell cycle. SNS-032, designed as a CDK2 inhibitor, also inhibits CDK7 and 9 and causes G_2_ cell cycle arrest [160,161]. CDK2 is necessary for progression through the G_1_/S checkpoint through its interaction with cyclin E and is necessary for progression through the S phase through its interaction with cycle A [162,163]. CDK7 and 9 play critical roles in transcription initiation and elongation and expression of both was decreased with treatment of UKF-NB-3 and IMR-32 neuroblastoma cells with SNS-032 [164]. However, most of the drug’s effects were due to cytotoxicity as opposed to a decrease in proliferation. CDK2 has also been targeted using miRNA and siRNA. miR-885-5p is a miRNA that downregulates CDK2. Both miR-885-5p and si*CDK2* inhibited neuroblastoma proliferation in a number of cell lines [165]. A specific inhibitor targeting CDK7, THZ1, has also been examined in neuroblastoma. In *MYCN*-driven neuroblastoma cells, THZ1 led to cell cycle arrest. Additionally, in *MYCN*-amplified neuroblastoma xenograft tumors in mice, cell cycle arrest and a decrease in markers associated with transcription were observed [166].

The genes encoding multiple proteins involved at cell cycle checkpoints are targets for a group of enzymes that remove acetylate groups from histones, termed histone deacetylases (HDACs). The balance between histone acetylation and deacetylation determines chromatin configuration, which determines the amount of gene transcription that can occur. Histone acetylation causes the chromatin to have an open configuration, allowing for transcription, whereas histone deacetylation compacts chromatin and blocks transcription. Genes that are repressed by HDACs ore often tumor suppressors, cell-cycle inhibitors, and inducers of apoptosis. A variety of HDAC inhibitors have been developed as potential treatments for cancer. In the Pediatric Preclinical Testing Program, vorinostat, a nonselective HDAC inhibitor, yielded significant growth inhibition in four neuroblastoma cell lines [167]. However, in neuroblastoma xenografts, vorinostat treatment did not lead to any objective responses. Other nonselective HDAC inhibitors—sodium butyrate, suberoylanilide hydroxamic acid, and trichostatin A—induced cell cycle arrest in the G_2_/M phase in neuroblastoma cells [168]. BL1521 is another HDAC inhibitor that caused G_1_ arrest along with an increase in the CKI P21, decreased CDK4, and RB hypophosphorylation [169,170]. The expression of HDAC8, a specific protein within the family, correlates with advanced disease and poor outcome in neuroblastoma [171]. By knocking down HDAC8 using siRNA, neuroblastoma cell proliferation decreased. The small molecule inhibitor of HDAC8, PCI-48012, decreased cell proliferation in neuroblastoma both *in vitro* and *in vivo* [172]. HDAC5 also promotes neuroblastoma cell proliferation and has been targeting using siRNA, yielding a decrease in proliferation [173]. However, no small molecule inhibitor specific for HDAC5 has been used in neuroblastoma. Other specific HDAC inhibitors also have efficacy in neuroblastoma, but through mechanisms other than a change in cell proliferation—HDAC1 and HDAC2 inhibitors induce differentiation, HDAC6 inhibitors decrease migration and invasion, and HDAC10 inhibitors decrease cell survival by autophagy [174,175,176]. Vorinostat has been examined in a phase I clinical trial in children with recurrent solid tumors, including two with neuroblastoma, and found to be well-tolerated [177]. None of the patients had objective responses to vorinostat alone, but one of the patients with neuroblastoma had a complete response to a combination of vorinostat and 13-cis retinoic acid. A number of other HDAC inhibitors are currently in pediatric clinical trials.

A kinome screen with RNAi found that loss of checkpoint kinase 1 (CHK1) was cytotoxic with ≥50% growth inhibition in neuroblastoma cells but no inhibition in normal somatic cells [178]. Given that it only decreases growth in the tumor cells, this is a promising therapeutic target. CHK1 regulates the G_1_/S and G_2_/M checkpoints and is expressed at a significantly higher level in *MYCN*-amplified compared to *MYCN*-unamplified neuroblastoma [178]. The small molecule checkpoint kinase inhibitors SB218078 and TCS2312 decreased proliferation of neuroblastoma cells. PF-00477736 is another CHK1 inhibitor that has been demonstrated to decrease tumor growth in mouse neuroblastoma xenografts. A phase I clinical trial was begun for PF-00477736, but was terminated for business reasons. Multiple clinical trials examining the CHK1 inhibitor LY2606368 in adults are in progress, but this drug has not yet been examined in neuroblastoma. A summary of drugs discussed in this section is available in Table 3.

**Table 3:** Regulators of the cell cycle affecting proliferation in neuroblastoma and the drugs that target them. Mechanism of action for each drug and stage of development in neuroblastoma is listed along with references.

Target Protein	Drug	Mechanism of Action	Stage of Development	References
ODC	DFMO	Binds/inhibits ODC irreversibly	Clinical trial	[146,151,152,155,156,157,158,159]
CDK4/6	Ribociclib/LEE011	Inhibits CDK4 and 6	Clinical trial	[148]
	Palbociclib/PD-0332991	Inhibits CDK4 and 6	*In vitro*	[149,150]
CDK2/7/9	SNS-032	Inhibits CDK2, 7 and 9	*In vitro*	[160,161,164]
CDK2	si*CDK2*	Double-stranded RNA cleaves *CDK2* mRNA	*In vitro*	[165]
	miR-885-5p	Single-stranded RNA inhibits translation of *CDK2* mRNA	*In vitro*	[165]
CDK7	THZ1	Inhibits CDK7	*In vivo*	[166]
Nonselective HDACs	Vorinostat	Nonselectively inhibits HDACs	Clinical trial	[167,177]
	Sodium butyrate	Nonselectively inhibits HDACs	*In vitro*	[168]
	Suberoylanilide hydroxamic acid	Nonselectively inhibits HDACs	*In vitro*	[168]
	Trichostatin A	Nonselectively inhibits HDACs	*In vivo*	[168,179]
	BL1521	Nonselectively inhibits HDACs	*In vitro*	[169,170]
HDAC8	si*HDAC8*	Double-stranded RNA cleaves *HDAC8* mRNA	*In vivo*	[171]
	PCI-48012	Binds metal binding site/inhibits HDAC8	*In vivo*	[172]
HDAC5	si*HDAC5*	Double-stranded RNA cleaves *HDAC5* mRNA	*In vivo*	[173]
CHK1	SB218078	Binds ATP pocket/inhibits CHK1 competitively	*In vitro*	[178]
	TCS2312	Binds ATP pocket/inhibits CHK1 competitively	*In vitro*	[178]
	PF-00477736	Binds ATP pocket/inhibits CHK1 competitively	*In vivo*	[178]

## 6. Conclusions

In conclusion, transcription factors, kinases, and regulators of cell cycle checkpoints play a significant role in cell proliferation in neuroblastoma. Despite the poor prognosis associated with neuroblastoma, there is vast potential for therapeutic targets, many of which are still in the early stages of examination and development, and many more of which have yet to be discovered. These novel therapies have the potential to transform the treatment of neuroblastoma in the future.
